# mTOR-mediated phosphorylation of VAMP8 and SCFD1 regulates autophagosome maturation

**DOI:** 10.1038/s41467-021-26824-5

**Published:** 2021-11-16

**Authors:** Hong Huang, Qinqin Ouyang, Min Zhu, Haijia Yu, Kunrong Mei, Rong Liu

**Affiliations:** 1 College of Food Science and Technology, Nanjing Agricultural University, Nanjing, China; 2 School of Life Sciences, Nanjing Agricultural University, Nanjing, China; 3 National Center for International Research on Animal Gut Nutrition, Nanjing Agricultural University, Nanjing, China; 4 Jiangsu Collaborative Innovation Center of Meat Production and Processing, Nanjing Agricultural University, Nanjing, China; 5 Jiangsu Key Laboratory for Molecular and Medical Biotechnology, College of Life Sciences, Nanjing Normal University, Nanjing, China; 6 School of Pharmaceutical Science and Technology, Tianjin University, Tianjing, China

**Keywords:** Lysosomes, Autophagy, SNARE

## Abstract

The mammalian target of rapamycin (mTORC1) has been shown to regulate autophagy at different steps. However, how mTORC1 regulates the N-ethylmaleimide-sensitive protein receptor (SNARE) complex remains elusive. Here we show that mTORC1 inhibits formation of the SNARE complex (STX17-SNAP29-VAMP8) by phosphorylating VAMP8, thereby blocking autophagosome-lysosome fusion. A VAMP8 phosphorylation mimic mutant is unable to promote autophagosome-lysosome fusion in vitro. Furthermore, we identify SCFD1, a Sec1/Munc18-like protein, that localizes to the autolysosome and is required for SNARE complex formation and autophagosome-lysosome fusion. VAMP8 promotes SCFD1 recruitment to autolysosomes when dephosphorylated. Consistently, phosphorylated VAMP8 or SCFD1 depletion inhibits autophagosome-lysosome fusion, and expression of phosphomimic VAMP8 leads to increased lipid droplet accumulation when expressed in mouse liver. Thus, our study supports that mTORC1-mediated phosphorylation of VAMP8 blocks SCFD1 recruitment, thereby inhibiting STX17-SNAP29-VAMP8 complex formation and autophagosome-lysosome fusion.

## Abstract (web summary)

Autophagy relies on coordinated fusion of organelle membranes, although the interplay between the regulatory machinery is not well studied. Here, the authors show that SNARE complex formation is inhibited by mTORC1 phosphorylation of VAMP8, which prevents autophagosome-lysosome fusion.

## Introduction

Autophagy is a highly conserved cell degradation and homeostasis pathway through which damaged organelles in the cytoplasm, misfolded proteins, or intracellular pathogens are engulfed by autophagosomes^1–5^. Autophagy has essential roles in cellular physiology in mitigating metabolic stress, avoiding genomic damage, and restoring damage or loss during diverse developmental processes. Accordingly, defects in autophagy have been implicated in various diseases including cancers, neurodegeneration disorders, nonalcoholic fatty liver disease (NAFLD), and cardiovascular disorders^6–11^.

Autophagy comprises multiple stages including autophagy induction, autophagosome formation, autophagosome-lysosome fusion, and autophagic lysosome degradation^12,13^. The soluble N-ethylmaleimide-sensitive factor attachment protein receptor (SNARE) complex is the core machinery responsible for the autophagosome-lysosome fusion process^14^. The Qa, Qb, Qc, and R SNARE proteins form a four-helix bundle that stabilizes the complex to promote autophagosome-lysosome fusion^15^. During autophagosome-lysosome fusion, the autophagosome-localized Qa protein Syntaxin 17 (STX17) cooperates with synaptosome-associated protein 29 (SNAP29, Qbc) and the lysosome-localized R protein-vesicle associated membrane protein 8 (VAMP8) to promote fusion^14^. The O-GlcNAcylation of SNAP29 attenuates the interaction between SNAP29 and STX17^16^. HDAC2 acetylated STX17 controls autophagosome maturation^17^. STX17 is reported to be phosphorylated by TBK1 for its translocation from the Golgi to its pre-autophagosomal structure (mPAS)^18^.

Mammalian target of rapamycin (mTORC1) is a kinase regulating metabolism, cell growth, and autophagy. It negatively modulates autophagy at both early and late stages^19–22^. mTORC1 inhibits autophagosome formation by phosphorylating unc-51-like autophagy-activating kinase 1 (ULK1), ATG13, ATG14, and WIPI2^23–25^. mTORC1 is also known to phosphorylate the tethering proteins UVRAG and Pacer to impair autophagosome maturation^26,27^, and mTORC1 phosphorylates the transcription factor EB (TFEB) to control autophagy and lysosome biogenesis at the transcriptional level^28,29^.

Here, we observed a significant decrease in mTORC1-mediated phosphorylation of the SNARE component VAMP8 upon culture in (autophagy-inducing) starvation conditions and showed that the dephosphorylated form of VAMP8 strongly promotes STX17-SNAP29-VAMP8 complex formation. Further, we established that the SM-like protein SCFD1 is required for autophagosome-lysosome fusion, specifically showing that SCFD1’s interaction with VAMP8 is responsible for its recruitment to autolysosomes. Stimulation of autophagy promoted the interaction between SCFD1 and VAMP8, thereby promoting STX17-SNAP29-VAMP8 complex formation and autophagosome-lysosome fusion. Correspondingly, studies in mice showed that VAMP8 phosphorylation both inhibit autophagosome-lysosome fusion and disturbs lipid metabolism in the liver.

## Results

### mTORC1 phosphorylates VAMP8 at T48

**Fig. 1:**
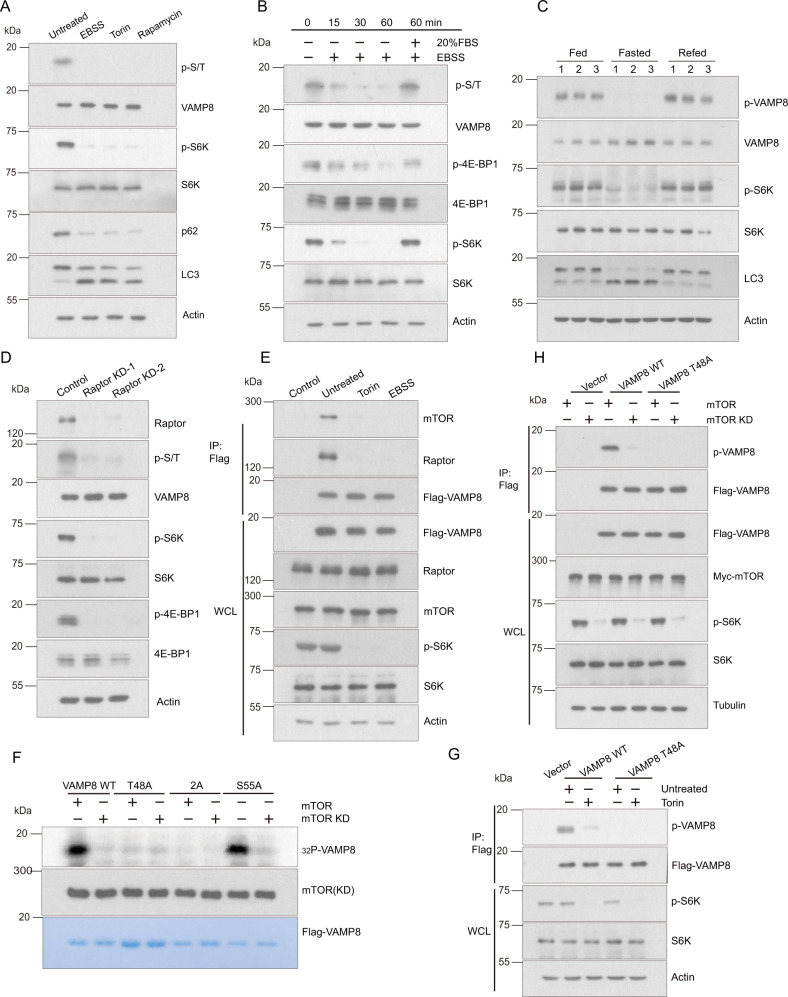
mTORC1 phosphorylates VAMP8 at T48. **A** The extent of VAMP8 phosphorylation decreased upon inhibition of mTORC1. Protein lysates were prepared from HEK293T cells cultured in rich medium (untreated), EBSS medium, or that treated with Torin1 or rapamycin. Phosphorylated VAMP8 was blotted using antibodies against anti-phospho-Ser/Thr. Actin, LC3, p62, S6K, and p-S6K were detected by the indicated endogenous antibodies. **B** Nutrient-stress-induced VAMP8 dephosphorylation in a time-dependent manner. HEK293T cells were stimulated with EBSS for 15, 30, or 60 min or cultured with complete medium for 2 h after 1 h starvation. The VAMP8 phosphorylation level was detected using anti-phospho-Ser/Thr antibodies. 4EBP1, p4EBP1, S6K, pS6K were detected by the indicated endogenous antibodies. **C** Fasting decreased VAMP8 phosphorylation in mice liver. Mice were fed with normal diet (Fed), fasted, or refed after fasting. Western blot was employed to analyze the indicated protein levels. **D** Raptor knockdown inhibits VAMP8 phosphorylation. Raptor expression was inhibited by transfection of HEK293T cells with anti-Raptor siRNA. VAMP8 phosphorylation level was detected by western blot. **E** mTORC1 associates with VAMP8 in a nutrient-depended manner. HEK293T cells were transfected Flag-VAMP8, and the interaction of Flag-VAMP8 and mTORC1/Raptor was analyzed by immunoprecipitation under full medium or EBSS/Torin treatment. The mTOR/Raptor level was analyzed by anti-mTOR/Raptor antibody. **F** mTORC1 phosphorylates VAMP8 T48 in vitro. Flag-VAMP8^WT^/VAMP8^T48A^/VAMP8^2A^/VAMP8^S55A^ recombinant protein was purified from *E. coli* and incubated with purified mTORC1 or an mTOR^D2357E^ (Kinase Dead) mutant. The phosphorylation level was detected by γ-^32^P-ATP autoradiography. **G** The specificity of the antibody for the T48-phosphorylated form of VAMP8. Flag-VAMP8^WT^/VAMP8^T48A^ proteins were pulled down using Flag beads and incubated with purified mTORC1 or mTORD^2357E^. The VAMP8 phosphorylation level was detected with anti-phospho-VAMP8(T48) antibodies. **H** Vector control or Flag-VAMP8^WT^/VAMP8^T48A^ was co-expressed with either Myc-tagged mTORC1 or mTOR^D2357E^ in HEK293T cells. Twenty-four hours after transfection, Flag-tagged proteins were purified by Flag beads and the VAMP8 phosphorylation level was detected by anti-phospho-VAMP8(T48) antibodies.

mTORC1 is a master kinase that is part of the upstream pathway of autophagy. VAMP8 has been identified as the R-SNARE protein controlling autophagosome-lysosome fusion^14^. Both mTORC1 and VAMP8 are resident proteins on lysosomes and function in autophagy. To investigate potential relationships between mTORC1 and VAMP8, we first generated a Flag-VAMP8 stable HEK293T cell line, which we exposed to various stimuli and assessed based on immunoblotting analyses. Phosphorylation of VAMP8 dramatically decreased when autophagy was induced by culture with starvation medium Earle’s Balanced Salt Solution (EBSS) or adding mTOR inhibitor Torin or rapamycin to the cultured cells (Fig. 1A). Also, the phosphorylation level of VAMP8 decreased in a time-dependent manner when the cells were treated with EBSS (Fig. 1B), and reduction of the extent of VAMP8 phosphorylation could be rescued by adding 20% fetal bovine serum (FBS) back into the medium (Fig. 1B). Consistent with this, the phosphorylation level of VAMP8 was decreased in mice liver when mTOR activity was inhibited by fasting (Fig. 1C). Knockdown of Raptor, a subunit of the mTORC1 complex, consistently led to reduced VAMP8 phosphorylation (Fig. 1D). These results suggest that VAMP8 can be phosphorylated by mTORC1. In addition, VAMP8 interacted with mTORC1 under nutrient rich conditions, whereas this was not observed for the Torin or EBSS treated cells (Fig. 1E).

We then wondered if mTORC1 phosphorylates VAMP8 directly, so we conducted in vitro kinase assays. VAMP8 was phosphorylated by mTOR in vitro, suggesting that VAMP8 is a direct substrate of mTOR (Fig. 1F). Next, we performed mass spectrometry (MS) analysis and identified two candidate phosphorylation sites of VAMP8: T48 and S55 (Figure S1). To explore if these sites are essential for VAMP8 phosphorylation, we generated single site (VAMP8^S55A/T48A^) or double site (VAMP8^2A^) dephosphorylation mimic VAMP8 variants. Our in vitro kinase assays showed that VAMP8^WT^ and VAMP8^S55A^, but not VAMP8^T48A^ or VAMP8^2A^, were phosphorylated by mTORC1 in vitro, indicating that mTORC1 directly phosphorylates the T48 residue of VAMP8 (Fig. 1F). To provide biochemical verification of the MS results, we also generated a phospho-specific antibody against VAMP8 T48. Consistent with our MS-based findings, the VAMP8^T48A^ mutation totally abolished VAMP8 phosphorylation with or without Torin treatment (Fig. 1G). To determine if mTORC1 activity is essential for phosphorylation of VAMP8 at T48, a kinase dead mutant of mTORC1 was used alongside Torin to inhibit mTOR activity; no VAMP8 phosphorylation was observed in the assays containing the kinase-dead mTOR variant with or without Torin to inhibit mTOR activity (Fig. 1H)^30^. Consistently, the VAMP8^T48A^ mutant was not phosphorylated by mTOR (Fig. 1H). Taken together, these results support that mTORC1 phosphorylates VAMP8’s T48 residue.

### VAMP8 phosphorylation negatively regulates autophagosome-lysosome fusion

**Fig. 2:**
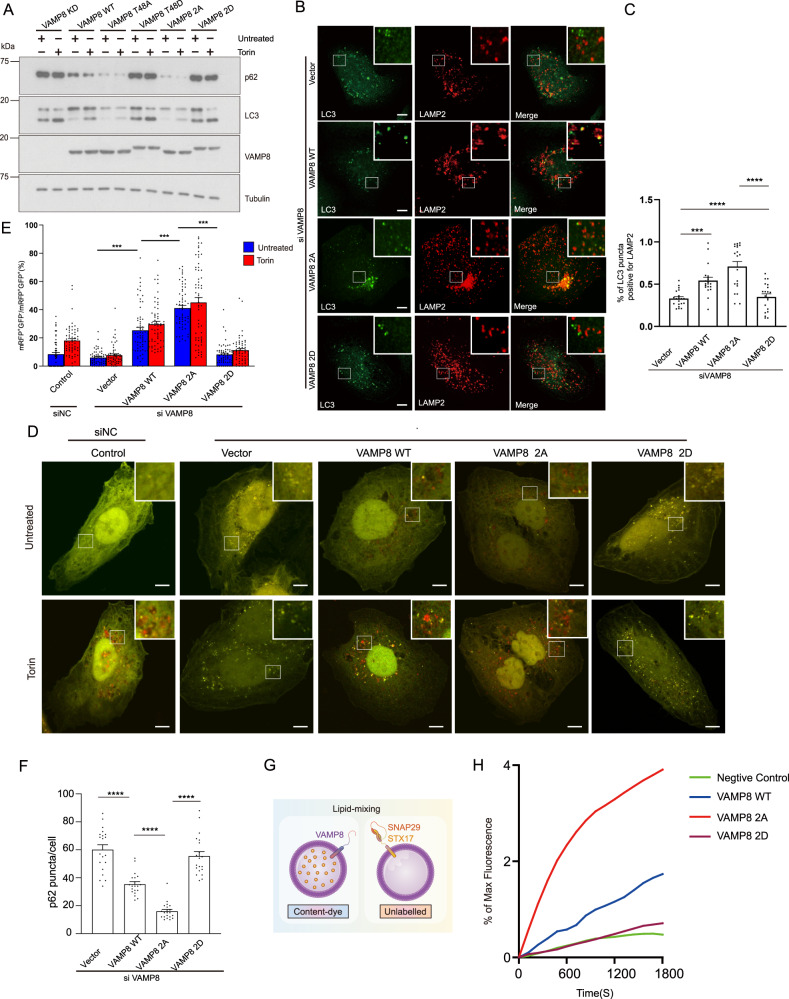
VAMP8 phosphorylation inhibits autophagosome-lysosome fusion. **A** VAMP8 phosphorylation blocks autophagic flux. VAMP8 deficient U2OS cells reconstituted with the VAMP8^WT^, VAMP8^T48A^, VAMP8^T48D^, VAMP8^2A^, or VAMP8^2D^ proteins. Cells were cultured in full medium, with or without Torin treatment. LC3 and p62 levels were detected by western blot. **B** VAMP8 phosphorylation impairs LC3-LAMP2 localization. VAMP8-deficient, Torin-treated U2OS cells complemented with VAMP8^WT^, VAMP8^2A^, or VAMP8^2D^ proteins, were stained with anti-LC3/LAMP2 antibodies and analyzed by immunofluorescence microscopy. Scale bar 5 μm. **C** Quantification of LC3- LAMP2 co-localization in **B**. Statistical significance was assessed by comparing different VAMP8 variants. One-way ANOVA (Values are shown as means ± SEM. *n* = 20., ***p < 0.001., ****p < 0.0001). **D** mRFP-GFP-LC3 assay in U2OS cells. mRFP-GFP-LC3 was expressed in U2OS cells (control siRNA, VAMP8 KD/rescued by vector, VAMP8^WT^, VAMP8^2A^, or VAMP8^2D^) and analyzed by confocal microscopy. Cells were cultured in full medium with or without Torin treatment. Scale bar 5 µm. **E** Quantitative analysis of the relative mRFP^+^-GFP^-^-LC3 puncta, as indicators of autophagosome maturation. One-way ANOVA (Data are shown as the mean ± SEM. ****p < 0.0001., *n* = 60). **F** VAMP8 phosphorylation blocks p62 degradation. Quantitative analysis of p62 immunofluorescence in VAMP8-deficient, Torin-treated U2OS cells complemented with VAMP8^WT^, VAMP8^2A^, or VAMP8^2D^ proteins. For quantification, the number of p62 puncta per cells were counted. One-way ANOVA (Data are shown as the mean ± SEM. ****p < 0.0001, *n* = 20). **G** Schematic for the experimental procedures used in the reconstituted fusion reactions. Liposomes were reconstituted with the indicated SNAREs to mimic autophagosome-lysosome fusion in vitro. **H** Fusion of the reconstituted proteoliposomes with phospho-VAMP8 blocks SNARE-dependent membrane fusion in vitro. Lipid mixing of the reconstituted liposome fusion reactions containing wild-type (WT) t-SNAREs (STX17-SNAP29) and WT VAMP8 or the indicated VAMP8 variants. t-SNARE (5 μm) and v-SNARE (1.5 μm) were added in each fusion reaction system. The fusion reactions were measured using NBD-fluorescence-based lipid mixing assays. VAMP8 CD (20 µM) was added at the beginning of the preincubation as a negative control. Fusion data are presented as a percentage of the maximum fluorescence change.

To test any effects of VAMP8 phosphorylation on autophagy, we complemented VAMP8 knockdown U2OS cells with wild type VAMP8, VAMP8^T48A^, or VAMP8^T48D^ as well as the VAMP8^2A^ (T48A/S55A; dephosphorylation mimic) or VAMP8^2D^ (T48D/S55D; phosphomimic) variants of VAMP8. VAMP8 knockdown inhibited the degradation of LC3 and p62 (Fig. 2A). More LC3 and p62 accumulated in cells expressing the VAMP8^T48D^ and VAMP8^2D^ variant compared to cells with VAMP8^WT^. In direct contrast, the extent of LC3 and p62 degradation was obviously greatest in cells expressing the dephosphorylation mimic VAMP8^T48A^ and the VAMP8^2A^ variant, suggesting that VAMP8 dephosphorylation promotes autophagic flux (Fig. 2A). To test whether VAMP8 phosphorylation affects autophagosome-lysosome fusion, we examined the effect of VAMP8 depletion on LC3 (indicate autophagosome) and LAMP2 (indicate lysosome) co-localization after Torin stimulation. When VAMP8 was depleted, co-localization between autophagosomes and lysosomes was significantly reduced. Further, the expression of a VAMP8 dephosphorylation mimic mutant (VAMP8^2A^), but not of the VAMP8 phosphorylation mimic mutant (VAMP8^2D^), rescued LC3-LAMP2 co-localization in VAMP8-depleted cells (Fig. 2B, C).

To determine which autophagy steps are affected by VAMP8 phosphorylation, we used mRFP-GFP-LC3 as a reporter^1^; in this system GFP fluorescence is quenched in acidic organelles like lysosomes and yellow puncta (mRFP+ , GFP+ ) represent autophagosomes while red puncta (mRFP+ , GFP−) represent autolysosomes. The ratio of red/yellow puncta indicates the autophagosome maturation rate. The mRFP-GFP-LC3 assay results also showed that VAMP8 phosphorylation inhibits autophagosome-lysosome fusion, as shown by the increased proportion of yellow puncta in VAMP8^2D^ overexpressing cells stimulated with Torin (Fig. 2D, E). In contrast, VAMP8^2A^ mutant significantly increased the ratio of autolysosomes (red puncta) (Fig. 2D, E). Quantification of the endogenous p62 puncta in these cells also showed that p62 degradation was impaired in VAMP8^2D^ cells; in contrast, p62 degradation was accelerated in cells expressing VAMP8^2A^ (Fig. 2F).

We then tested if VAMP8 phosphorylation directly inhibits fusion in liposome lipid mixing assays wherein liposomes were reconstituted with various VAMP8 to mimic lysosome in vitro (Fig. 2G). We observed that liposome fusion occurred more rapidly with the VAMP8^2A^ variant compared to VAMP8^WT^ (Fig. 2H). By contrast, no liposome fusion was observed in reactions containing the VAMP8^2D^ variant (Fig. 2H). Taken together, VAMP8 phosphorylation inhibits STX17-SNAP29-VAMP8 mediated autophagosome-lysosome fusion.

Mitophagy is a special form of autophagy that selectively eliminates damaged mitochondria^31,32^. When mitophagy is induced, Parkin is recruited to damaged mitochondria to initiate mitophagy. To assess whether VAMP8 phosphorylation impacts mitophagy, we performed mitochondria clearance assays. HeLa cells stably expressing mCherry-Parkin were co-transfected with different VAMP8 overexpression plasmids (VAMP8^WT^, VAMP8^2A^, or VAMP8^2D^), followed by the classic mitophagy inducer treatment of oligomycin and antimycin (OA). Mitochondrial proteins Tom20, Cox2, and Phb2 were efficiently degraded in control cells after 12 h of OA treatment (Supplemental Fig. 2, left, lanes 1–4). However, the degradation of Tom 20, Cox2, and Phb2 were largely blocked in the VAMP8 knockdown cells (Supplemental Fig. 2, left, lanes 5–8). Similar mitophagy defects were also observed in cells expressing VAMP8^2D^ (Supplemental Fig. 2, right, lanes 9–12). Complementation with VAMP8^WT^ rescued this phenotype (Supplemental Fig. 2, right, lanes 1–4), while complementation with VAMP8^2A^ actually accelerated the degradation of mitophagy substrates (Supplemental Fig. 2, right, lanes 5–8). Taken together, these results demonstrate that VAMP8 phosphorylation blocks macroautophagy and mitophagy.

### mTORC1-dependent VAMP8 phosphorylation blocks STX17-SNAP29-VAMP8 SNARE complex formation

**Fig. 3:**
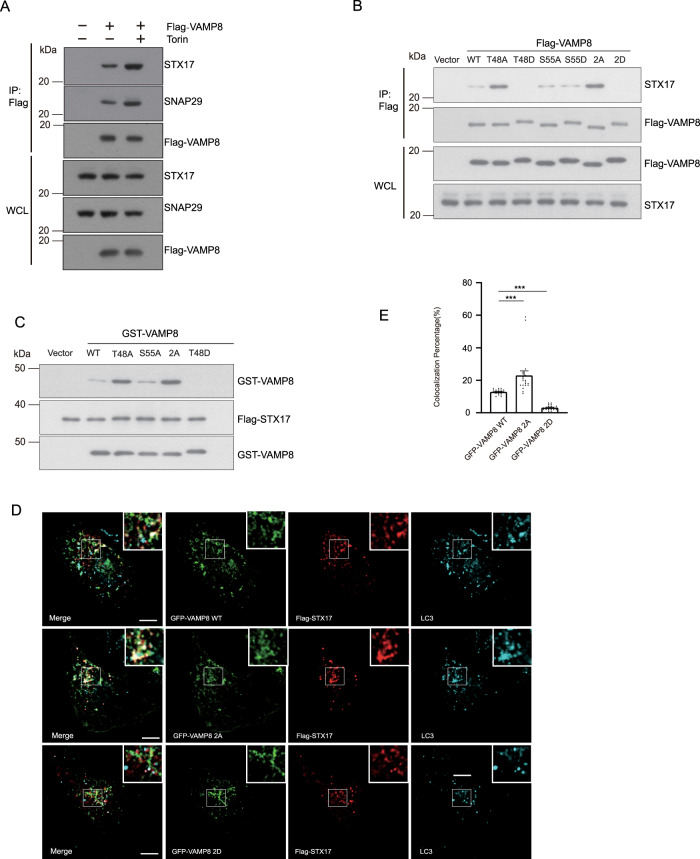
VAMP8 phosphorylation impairs SNARE complex formation. **A** Flag-Trap assays testing Flag-VAMP8 precipitation of endogenous STX17 and SNAP29 in Torin-treated or untreated control cells. Cell lysates from HEK293T cells transfected with Flag-VAMP8 were immunoprecipitated by Flag-beads with or without Torin treatment. STX17 and SNAP29 were detected by anti-STX17/SNAP29 antibody. **B** SNARE complex formation was clearly impeded upon disruption of mTORC1-mediated VAMP8 phosphorylation based on a single (T48D) mutation or a double (T48D, S55D) mutation. Flag-VAMP8^WT^ or VAMP8 variants (VAMP8^T48A^, VAMP8^T48D^, VAMP8^S55A^, VAMP8^S55D^, VAMP8^2A^, or VAMP8^2D^) were transfected into HEK293T cells. The interactions between transfected Flag-tagged proteins and endogenous STX17 were visualized by Flag co-immunoprecipitation and western blotting. **C** In vitro GST pull-down analysis using GST, GST-Tagged VAMP8^WT^, or the indicated GST-tagged VAMP8 variants, and Flag-STX17 recombinant proteins, followed by western blotting. **D** Markedly enhanced Flag-VAMP8^2A^ overlap with GFP-STX17 and endogenous LC3 compared with Flag-VAMP8^WT^. Immunofluorescence of U2OS cells expressing GFP-STX17 and Flag- VAMP8^WT^, VAMP8^2A^, or VAMP8^2D^ variant proteins. Scale bar, 10 µm. **E** Quantification for **E**. One-way ANOVA (Data are shown as the mean ± SEM. ***p < 0.001, *n* = 20).

Autophagosome-lysosome fusion is mediated by the SNARE complex STX17-SNAP29-VAMP8. To understand how VAMP8 phosphorylation by mTOR regulates autophagosome-lysosome fusion, we first tested whether mTORC1 inhibition affects SNARE complex formation. We treated cells with Torin and found that the interaction of VAMP8 with STX17 and SNAP29 increased upon mTORC1 inhibition (Fig. 3A).

To further investigate whether phosphorylation of VAMP8 regulates STX17-SNAP29-VAMP8 SNARE complex formation, we compared the interaction of VAMP8 mutants with STX17. VAMP8^T48A^ dramatically increased SNARE complex formation, whereas the VAMP8^S55A^ variant had little if any impact on SNARE complex formation (Fig. 3B). Further, VAMP8^T48D^ and VAMP8^2D^ blocked the fusion of autophagosomes and lysosomes, as well as the interaction between VAMP8 and endogenous STX17 (Fig. 3B). Recombinant VAMP8^T48A^ and VAMP8^2A^ dephosphorylation mimic variants, but not the VAMP8^S55A^ mutant, increased the extent of the interaction with STX17 (Fig. 3C). In contrast, the VAMP8^T48D^ variants abolish interaction with STX17 (Fig. 3C). Working with the crystal structure of STX17-SNAP29-VAMP8, T48 is positioned at layer +3, which is very close to the ionic layer, and T48 faces towards the inside of the SNARE complex (Supplemental Fig. 3). Consistent with the biochemical results described above (Fig. 3B, C), compared to VAMP8^WT^, the VAMP8^2A^ dephosphorylation mimic mutant displayed increased colocalization with STX17 and LC3 (Fig. 3D, E). In contrast, the VAMP8^2D^ phosphomimic mutant lost almost all localization with STX17 (Fig. 3D, E). Taken together, these data suggest that phosphorylation of VAMP8 by mTOR inhibits autophagosome-lysosome fusion.

### SCFD1 interacts with the STX17-SNAP29-VAMP8 SNARE complex

**Fig. 4:**
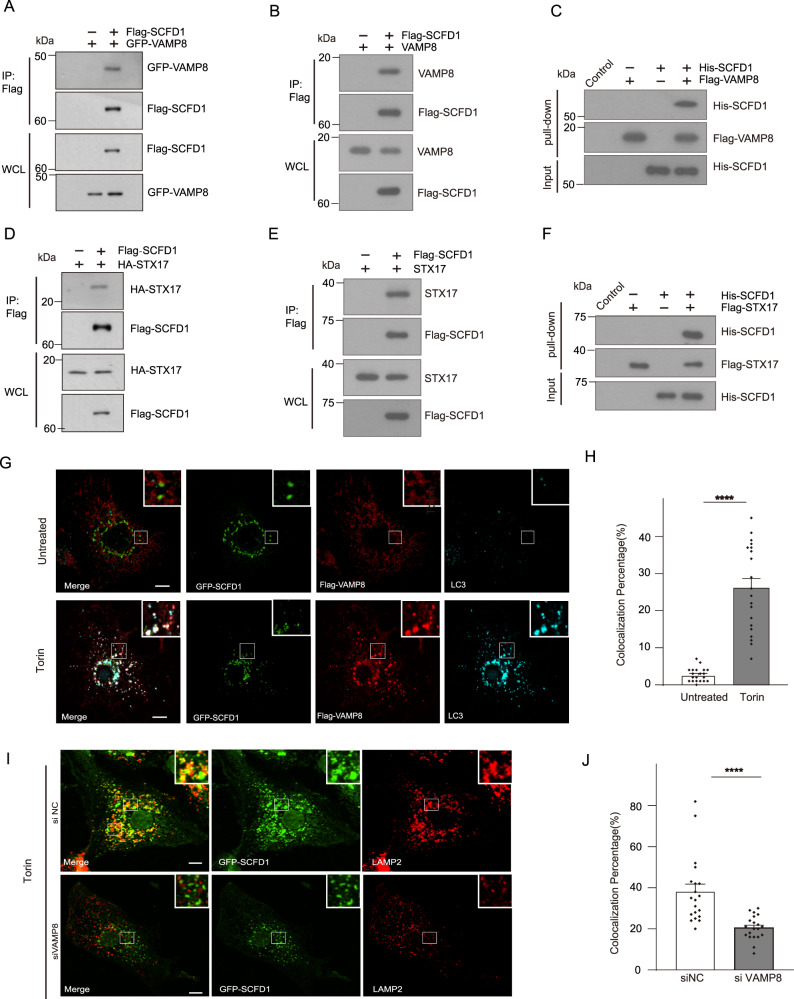
SCFD1 is an autophagic SNARE complex regulator. **A** Flag-Trap assays showing the interaction between SCFD1 and VAMP8 upon co-immunoprecipitation (co-IP) using HEK293T cells expressing Flag-SCFD1 and GFP-VAMP8. **B** Robust interaction between endogenous VAMP8 and Flag-SCFD1. Flag-tagged SCFD1 was transfected into HEK293T cells, Flag-beads were used to pull-down Flag-SCFD1, and VAMP8 was detected by anti-VAMP8 antibody. **C** Clear signals for Flag-VAMP8 were detected in pull-down analysis of recombinant His-SCFD1 and Flag-VAMP8. **D** Interaction between transfected Flag-tagged SCFD1 and HA-STX17, observed by Flag co-IP assay. **E** Robust interaction between Flag-tagged SCFD1 with endogenous STX17, observed in a co-IP assay. STX17 was detected by anti-STX17 antibody. **F** In vitro pull-down of purified recombinant Flag-STX17 with His-tagged SCFD1, showing the direct interaction between STX17 and SCFD1. **G** Immunofluorescence under confocal microscopy revealed the colocalization of GFP-SCFD1 with Flag-VAMP8 and endogenous LC3 in U2OS cells under Torin stimulation. Scale bar, 5 μm. **H** Quantification for Figure G. One-way ANOVA (Data are shown as the mean ± SEM. ****p < 0.0001, *n* = 20). **I** The extent of SCFD1/LAMP1 overlap was reduced upon siRNA-mediated knockdown of VAMP8. Immunofluorescence of endogenous LAMP1 and GFP-SCFD1 was observed in U2OS cells with the indicated siRNAs, with Torin treatment. Scale bar, 5 μm. **J** Quantification for **I**. One-way ANOVA (Data are shown as the mean ± SEM. ****p < 0.0001, *n* = 20).

We also examined VAMP8-associated proteins by tandem affinity purification (TAP) to investigate additional potential regulators of the STX17-SNAP29-VAMP8 SNARE complex. These assays used Tet-on Flag-VAMP8 cells (which express Flag tagged VAMP8 under the control of tetracyclin) and we found that SCFD1 has a robust interaction with VAMP8 (Supplemental Fig. 1B). To confirm the physical interaction between VAMP8 and SCFD1, we carried out co-immunoprecipitation assays in HEK293T cells. As expected, GFP-VAMP8 or endogenous Vamp8 was immunoprecipitated by Flag-SCFD1 (Fig. 4A, B). Moreover, purified recombinant Flag-VAMP8 could also pull down recombinant His-SCFD1 in vitro (Fig. 4C). These results support the genuine interaction of VAMP8 with SCFD1. We also tested if another autophagic SNARE protein, STX17, interacts with SCFD1. HA-STX17 or endogenous STX17 was immunoprecipitated by Flag-SCFD1 (Fig. 4D, E). Recombinant Flag-STX17 interacted with His-SCFD1 in vitro (Fig. 4F). We also tested the specificity of the SCFD1 and SNARE interaction. Flag-STX17 but not Flag-CD63 or Flag-LAMP1 interacted with endogenous SCFD1 (Supplemental Fig. 4A and B). Consistently, Flag-SCFD1 immunoprecipitated with endogenous STX17 and VAMP8 but not with CD63 (Supplemental Fig. 4C). These observations support the specificity of the interaction between SCFD1 and the STX17-SNAP29-VAMP8 complex.

Truncation variants of STX17 and VAMP8 were generated to support delineation of which functional domain mediated their interactions with SCFD1. We co-expressed Flag-tagged STX17 truncation constructs and GFP-tagged SCFD1 in HEK293T cells and found that deletion of the STX17 SNARE domain (162–223 aa) dramatically decreased SCFD1 binding (Supplemental Fig. 5).

SCFD1 is a Sec1/Munc18 (SM)-like protein that is conserved from yeast to mammals. SCFD1 functions with Stx5 in ER-Golgi trafficking^33,34^ and secretion, and loss of SCFD1 was shown to block the transport of extracellular matrix proteins from the ER to the Golgi^35^. There are no reports of SCFD1 functioning in autophagosome-lysosome fusion. To further investigate where in cells the VAMP8-SCFD1 interaction occurs we examined the location of SCFD1 in U2OS cells and noted that SCFD1 colocalized with autolysosomes (indicated by VAMP8 and LC3) in GFP-SCFD1 expressing cells cultured under starvation conditions (Fig. 4G, H). Immunostaining of VAMP8 knockdown cells revealed failed recruitment of GFP-SCFD1 to lysosomes, indicating that SCFD1 colocalization with lysosomes requires VAMP8 (Fig. 4I, J).

### SCFD1 promotes autophagosome-lysosome fusion

**Fig. 5:**
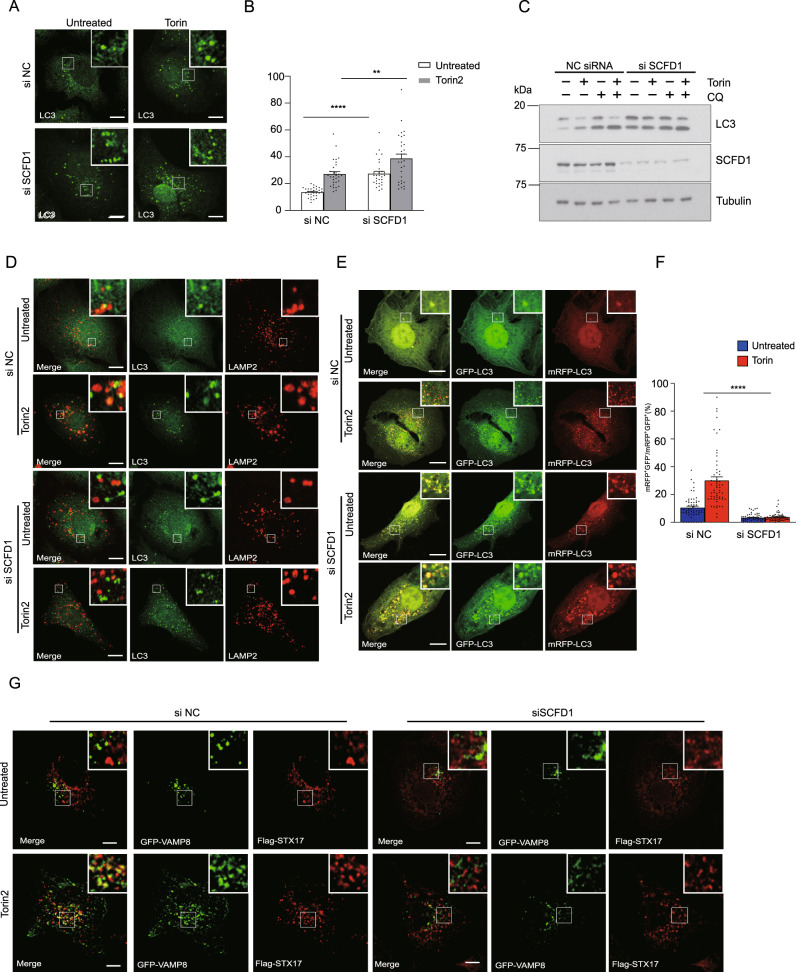
SCFD1 regulates autophagosome-lysosome fusion. **A** The number of LC3 puncta clearly increased upon SCFD1 knockdown. U2OS cells were transfected with indicated the siRNAs. Cells were cultured in full medium stimulated with Torin. LC3 puncta were detected using an LC3 antibody and observed via confocal microscopy. Scale bar, 10 μm. **B** Quantification of LC3 puncta per cell. One-way ANOVA (Data are shown as the mean ± SEM. **p < 0.01., ****p < 0.0001, *n* = 30). **C** SCFD1 knockdown blocks autophagy flux. U2OS cells transfected with either siSCFD1 or control siRNAs were incubated with full medium, full medium with Torin, or full medium with Torin and chloroquine (CQ). The LC3 was detected by western blotting. **D** The extent of LC3/LAMP2 overlap was reduced upon siRNA-mediated knockdown of SCFD1. Immunofluorescence of endogenous LC3 and Lamp2 was observed in U2OS cells with the indicated siRNAs, with or without Torin treatment. Scale bar, 10 μm. **E** SCFD1 depletion results in autophagosome accumulation. mRFP-GFP-LC3 assays performed in U2OS cells transfected with the indicated siRNAs, with or without Torin1 stimulation. Scale bar, 10 µm. **F** Quantification of data from part G. The ratio of GFP negative mRFP positive LC3 puncta versus both GFP and mRFP positive LC3 puncta. One-way ANOVA (Data are shown as the mean ± SEM. ****p < 0.0001, *n* = 58). **G** Localization of VAMP8 and STX17 is dependent on SCFD1. Immunofluorescence of U2OS cells expressing GFP-VAMP8 and Flag-STX17, transfected with indicated siRNAs, treated with or without Torin1. Scale bar, 5 µm. No overlap between STX17 and VAMP8 was detected upon siRNA-mediated knockdown of SCFD1.

To investigate if SCFD1 functions in autophagy we conducted experiments with siRNA-mediated SCFD1 knockdown in Torin-stimulated U2OS cells. SCFD1 knockdown led to a significant increase in LC3 accumulation (Fig. 5A, B). Autophagy flux assays also showed that autophagosome maturation was blocked in SCFD1 KD cells (Fig. 5C).

Consistently, co-localization between autophagosomes and lysosomes was significantly reduced in SCFD1 KD cells (Fig. 5D). We also conducted tandem mRFP-GFP-LC3 assays to monitor autophagy and found that compared to wild type cells, SCFD1 KD cells had significantly more yellow puncta, suggesting that SCFD1 depletion blocks autophagosome-lysosome fusion (Fig. 5E, F). Consistent with the results above, the extent of STX17 and VAMP8 colocalization decreased significantly in SCFD1 knockdown cells (Fig. 5G). These results establish that SCFD1 is required for autophagosome-lysosome fusion.

### Phosphorylation of VAMP8 by mTORC1 inhibits SCFD1-SNARE interaction

**Fig. 6:**
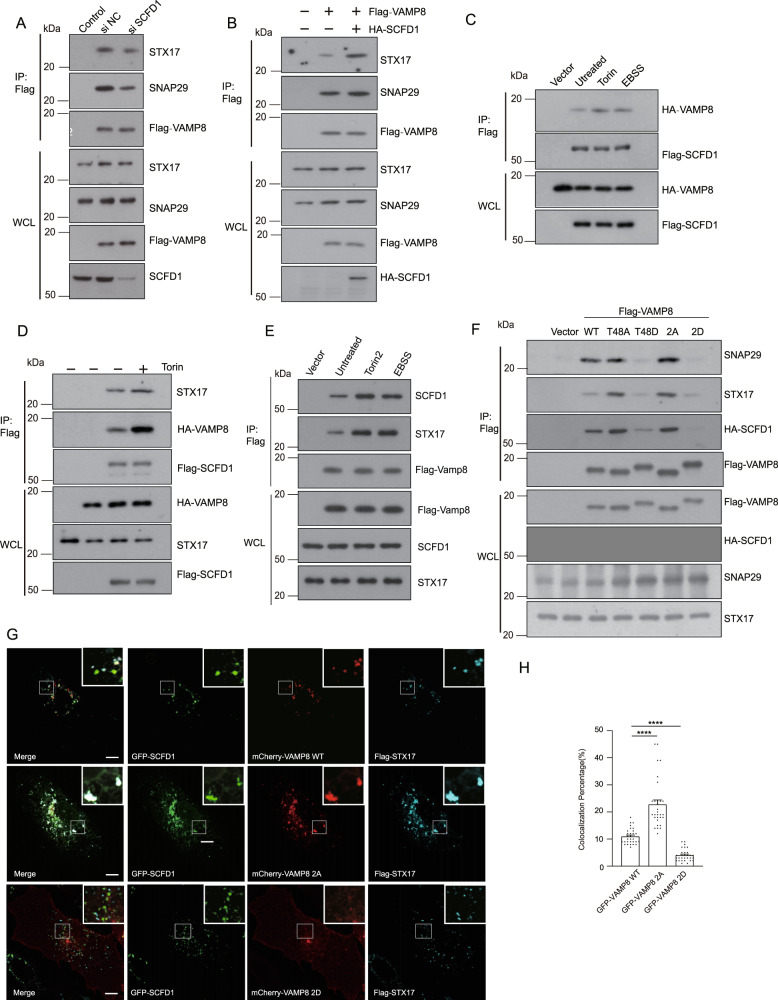
mTORC1 regulates the SCFD1-SNARE interaction. **A** Co-immunoprecipitation (co-IP) assays revealed that STX17-SNAP29-VAMP8 SNARE complex formation was decreased upon siRNA-mediated knockdown of SCFD1. HEK293T cells expressing Flag-VAMP8 were transfected with control siRNA or siSCFD1. STX17, SNAP29, and SCFD1 were detected by western blotting. **B** Robust enhancement of STX17-SNAP29-VAMP8 SNARE complex formation was detected upon overexpression of Flag-SCFD1 in HEK 293T cells. Flag-tagged VAMP8 was co-expressed with or without HA-SCFD1 after Flag co-IP; STX17 and SNAP29 were detected by western blotting. **C** Suppression of mTOR activation by Torin or EBSS treatment; the interaction between Flag-SCFD1 and HA-VAMP8 was increased. HEK293T cells were transfected with Flag-SCFD1 and HA-Vamp8. At 24 h post-transfection, cells were incubated with full medium, full medium with Torin, or starvation medium, followed by observation of the SCFD-VAMP8 interaction by Flag-immunoprecipitation. Co-IP assays revealed that the interaction of Flag-SCFD1 and HA-Vamp8/STX17 was robustly increased upon inhibition of mTOR. **D** The presence of mTOR inhibited the interaction between Flag-SCFD1 and GFP-VAMP8. Flag-SCFD1 and GFP-VAMP8 were co-expressed with Myc-tagged mTOR^WT^ in HEK293T cells. Co-IP was performed using Flag beads. **E** Stimulated autophagy by Torin or EBSS treatment; the interaction between Flag-VAMP8 and endogenous STX17/SCFD1 was increased. HEK293T cells were transfected with Flag-VAMP8. Co-IP assays revealed that the interaction of Flag-VAMP8 and STX17/SCFD1 was robustly increased upon inhibition of mTOR. **F** VAMP8 mutant variants mimicking mTOR phosphorylation (VAMP8^T48D^/ VAMP8^2D^) lost their capacity to interact with STX17/SNAP29/SCFD1. Individual VAMP8 mutations mimicking mTOR-mediated phosphorylation (VAMP8^T48D^/ VAMP8^2D^) or dephosphorylation (VAMP8^T48A^, VAMP8^2A^) were co-expressed with HA-SCFD1 to analyze their interaction(s) with STX17, SNAP29, and/or SCFD1. The STX17 and SNAP29 levels were detected by anti-STX17/SNAP29 antibody. **G** Colocalization of VAMP8^2A^ and SCFD1 was increased compared with VAMP8^WT^. U2OS cells stably expressing VAMP8^WT^ or mutants that mimicked (VAMP8^2A^) or resisted (VAMP8^2D^) phosphorylation were transfected with GFP-SCFD1/Flag-STX17 and then treated with or without Torin. Cells were fixed and subjected to immunofluorescence microscopy using GFP/Flag antibody. Scale bar, 5 μm. **H** Quantification for **F**. One-way ANOVA (Data are shown as the mean ± SEM. ****p < 0.0001, *n* = 30).

Given the fundamental role of the STX17-SNAP29-VAMP8 SNARE complex in autophagosome-lysosome fusion^14^, we examined the effects of SCFD1 on STX17-SNAP29-VAMP8 SNARE complex formation. STX17-SNAP29-VAMP8 complex formation decreased in SCFD1 KD HEK293T cells (Fig. 6A). In contrast, STX17-SNAP29-VAMP8 complex formation was dramatically increased over HEK293T cells upon SCFD1 overexpression (Fig. 6B). These results show that SCFD1 somehow regulates STX17-SNAP29-VAMP8 complex formation.

Having functionally linked SCFD1 to STX17-SNAP29-VAMP8 complex formation, we further studied whether the SCFD1-SNARE complex is regulated by any upstream signals during autophagosome-lysosome fusion. Specifically, we examined whether mTORC1 regulates the fusion activity of SCFD1. When mTORC1 was inhibited by Torin or EBSS treatment, the extent of the VAMP8-SCFD1 interaction significantly increased (Fig. 6C). This negative correlation between mTORC1-mediated VAMP8 phosphorylation and the extent of the VAMP8-SCFD1 interaction raises the possibility that the phosphorylation of VAMP8 by mTORC1 is involved in SCFD1-SNARE complex formation. Supporting this hypothesis, Torin or EBSS mediated autophagy stimulation led to increased Flag-SCFD1-VAMP8/STX17 interaction and increased Flag-VAMP8-STX17/SCFD1 interaction (Fig. 6D, E). Thus, mTOR activity regulates the SCFD1-SNARE interaction.

Further, we tested if the VAMP8 variant mutants inhibit the VAMP8-SCFD1 interaction. Compared with VAMP8^WT^, increased SCFD1 was co-precipitated with VAMP8^2A^. In contrast, the interaction between the phospho-mimic VAMP8^2D^ variant and SCFD1 was abolished (Fig. 6F). Immunofluorescence analysis also showed that GFP-SCFD1 had increased colocalization with the dephosphorylation mimic Flag-VAMP8^2A^ variant compared to Flag-VAMP8^WT^. In contrast, the extent of Flag-VAMP8^2D^ and GFP-SCFD1 colocalization was significantly decreased (Fig. 6G, H). Taken together, these results support that mTORC1-mediated VAMP8 phosphorylation represses SCFD1-VAMP8 interaction and STX17-SNAP29-VAMP8 complex formation.

### Livers of mice constitutively expressing the phosphomimic VAMP8 variant display dramatic accumulation of lipid droplets

**Fig. 7:**
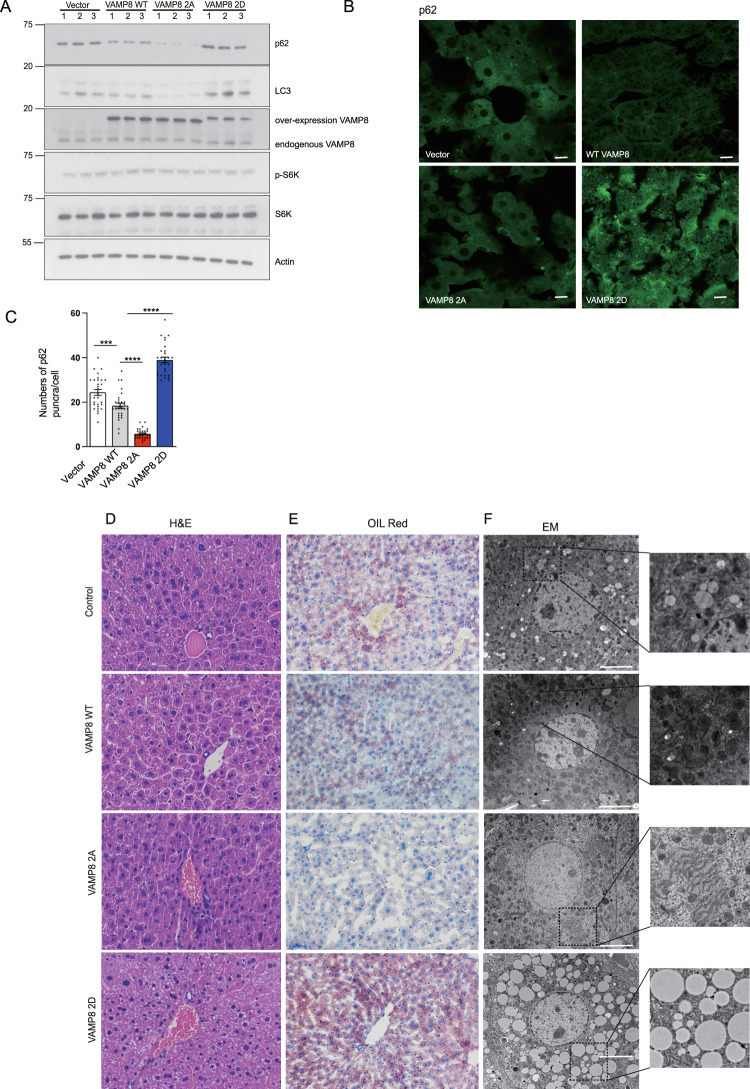
VAMP8 phosphorylation regulates lipid homeostasis in liver. **A** VAMP8 mutations mimicking dephosphorylation (VAMP8^2A^) accelerated p62 degradation compared with VAMP8^WT^ in mice livers. In contrast, p62 was dramatically accumulated in liver tissue lysates expressing the phosphorylation mimic VAMP8^2D^ variant. The p62 levels from liver tissues from AAV-VAMP8^WT^, AAV-VAMP8^2A^, or AAV-VAMP8^2D^ injected mice were detected by western blotting. **B** Representative images of immunofluorescence staining for p62 in liver sections from mice liver specific expressing VAMP8, VAMP8^2A^, or VAMP8^2D^. Scale bar, 100 µm. **C** Quantification of p62 puncta per cell are shown. One-way ANOVA (Data are shown as the mean ± SEM. ***p < 0.001. ****p < 0.0001, *n* = 30). **D** Representative images of HE stained liver sections from mice with liver-specific expression of the VAMP8^WT^, VAMP8^2A^, or VAMP8^2D^ proteins. Scale bar, 100 μm. 200×, *n* = 8/group. **E** Oil red O staining of liver sections from the indicated mouse livers. Scale bar, 100 µm. 200×, *n* = 8/group. **F** Transmission electron microscopy from the indicated mouse livers. Scale bar, 5 µm. 6000×, *n* = 8/group.

Many lines of evidence highlight the functional impacts of autophagy in lipid homeostasis^36,37^, and we thus investigated whether VAMP8-phosphorylation-regulated autophagosome-lysosome fusion contributes to lipid homeostasis in vivo. We injected mice with adeno-associated virus 9(AAV9) to drive the liver-specific expression of the VAMP8^WT^, VAMP8^2A^, or VAMP8^2D^ proteins. After injection, we examined whether VAMP8 phosphorylation regulates autophagy in vivo. Compared to the VAMP8^WT^ animals, the p62 level was clearly decreased in VAMP8^2A^ livers but substantially increased in VAMP8^2D^ livers (Fig. 7A), findings consistent with our earlier assays with the VAMP8^WT^, VAMP8^2A^, and VAMP8^2D^ U2OS cell lines. These trends were further confirmed by immunostaining against p62 in mouse livers (Fig. 7B, C), supporting that VAMP8 phosphorylation blocks autophagosome-lysosome fusion in vivo.

The results of hematoxylin-eosin (HE) staining (Fig. 7D), oil red O staining (Fig. 7E), and electron microscope (EM) (Fig. 7F) all showed that lipid droplets were dramatically accumulated in the livers of the VAMP8^2D^ expressing mice compared to the VAMP8^WT^ mice. In contrast, significantly fewer lipid droplets were detected in the VAMP8^2A^ livers (Fig. 7C–E). Together, these results demonstrate that VAMP8 phosphorylation is essential for maintaining lipid hemostasis in vivo.

## Discussion

mTORC1 strictly controls multiple steps of autophagy, including autophagosome formation and autophagosome-lysosome fusion^4,38–40^. Here, we show that VAMP8 is a direct substrate of mTORC1. Phosphorylation of VAMP8 inhibits autophagosome-lysosome fusion by disrupting STX17-SNAP29-VAMP8 SNARE complex formation. Furthermore, we identified a new regulator of autophagosome-lysosome fusion: SCFD1. With the inhibition of mTORC1, the phosphorylation of VAMP8 by mTORC1 decreases, which in turn promotes the interaction of VAMP8 with both SCFD1 and the STX17-SNAP29 binary SNARE complex, ultimately promoting autophagosome-lysosome fusion.

Recently, autophagic SNARE complexes have been identified to drive autophagosome-lysosome fusion. SNARE proteins are divided into four sub-families: Qa-, Qb-, Qc-, and R-SNAREs. The autophagosome localized STX17 (Qa) and SNAP29 (Qbc), along with the lysosome anchored VAMP8 (R), assemble into four-α-helical bundles to stabilize the SNARE complex. Formation of the SNARE complex is precisely controlled by upstream signals: SNAP29 is O-GlcNAcylated by O-linked β-N-acetylglucosamine (O-GlcNAc) transferase 1, as an O-GlcNAc-defective SNAP29 mutation variant promotes autophagic SNARE formation^41^. Similarly, STX17 is acetylated by CREBBP/CBP to inhibit SNARE complex formation^17^. STX17 is phosphorylated by TBK1, which promotes its translocation from the Golgi to mPAS^18^, and mTORC1 was recently shown to phosphorylate CREBBP to inhibit STX17 acetylation^17^. Under nutrient-rich conditions, mTORC1 interacts with VAMP8 and phosphorylates it. Compared to dephosphorylated VAMP8, phosphorylated VAMP8 undergoes reduced interactions with the SNARE proteins STX17 and SNAP29, ultimately resulting in failed autophagosome-lysosome fusion. Given that autophagy can be induced by many stimuli and in different contexts, it is not yet clear if other post-translational modifications of these SNARE proteins may also affect autophagosome-lysosome fusion.

SCFD1 is a Sec1/Munc18 (SM)-like protein with a high degree of evolutionary conservation. The SM proteins were first isolated in yeast and *C. elegans* by genetic screening^42,43^. Membrane fusion activity is blocked in the absence of SM proteins^44–46^. Neuro SNARE has been well-studied and is known to influence neurotransmitter release. During this process, syntaxin-1/SNAP-25 (both in the plasma membrane), and the synaptic-vesicle-localized synaptobrevin assemble into the trans-SNARE complex, which drives the fusion of vesicles and the plasma membrane. This complex contains a four α-helix bundle with a polar layer comprising three glutamines and one arginine^47^. The synaptic SM protein Munc18-1 initially finds and interacts with synataxin-1. Munc18-1 clasps the four α-helix bundle and stabilizes the trans-SNARE complex during neurotransmitter secretion^48–50^. Deletion of the SM protein Munc18-1 in mice leads to extremely severe neurotransmitter secretion defects^51^. In humans, mutations in Munc18-1 can cause early infantile epileptic encephalopathy^52^. Here, we demonstrated that SCFD1 interacts with and regulates the formation of the STX17-SNAP29-VAMP8 complex: the efficiency of SNARE complex assembly was dramatically decreased in cells lacking functional SCFD1 and the autophagosome-lysosome fusion rate is was also clearly blocked. Autophagic SNARE promotes autophagosome-lysosome vesicle fusion. Upon autophagy induction, SCFD1 is recruited to autolysosomes in a VAMP8-dependent manner. Given that SCFD1 is an SM-like protein, it seems plausible that SCFD1 may stabilize the STX17-SNAP29-VAMP8 trans-SNARE complex. It is also possible that SCFD1 restricts the SNARE complex into a specific cellular region during autophagosome-lysosome fusion or prevents SNARE complex disassembly.

The crystal structure of the neuro SNARE complex comprising syntaxin1, synaptobrevin, and SNAP25 was solved in 1998^53,54^. This complex forms a four-helix bundle that retains a highly twisted structure. The autophagic SNARE STX17-SNAP29-VAMP8 structure has recently been reported^55,56^. It is therefore likely that insights about the SCFD1-SNARE complex from structural biology will facilitate further elucidation of SCFD1 functions in coordinating SNARE complex formation and impacts on autophagosome-lysosome fusion.

Autophagy is essential for hepatic lipid hemostasis^57–59^. Our results from mice with liver-specific VAMP8 and VAMP8 mutant expression demonstrate that VAMP8 phosphorylation affects liver lipid metabolism, possibly by regulating the fusion of autophagosomes and lysosomes. Liver lipid hemostasis disorder is seen in many liver diseases, including non-alcoholic fatty liver disease (NAFLD) and alcoholic fatty liver disease (AFLD), among others^60,61^. Viewed in this context, regulation of the phosphorylation status of VAMP8 could be a promising strategy for developing therapeutics for the management of liver lipid accumulation.

## Methods

### Cell culture

HEK293T, Hela, and U2OS cells were cultured in Dulbecco’s Modified Eagle’s Medium (DMEM, SH30022.01B, Hyclone) supplemented with 10% fetal bovine serum (FBS) and 1% penicillin-streptomycin (15140163, Gibco) at 37 °C and 5% CO_2_. To induce autophagy, cells were treated with Earle’s Balanced Salt Solution (EBSS) or Torin1. Cells were incubated with chloroquine (C6628, Sigma) to block autophagy.

### Transient transfection and RNA interference

Transient transfection of plasmids was performed with Lipofectamine3000 (L3000075, Life Technologies) transfection reagent for 24–48 h before harvesting. For gene silencing, U2OS or HeLa cells were grown for 24 h to reach 40% density, then siRNAs were delivered by lipofectamine RNAiMAX (13778150, Life Technologies) transfection reagent for 48–72 h before harvesting.

### Immunoblotting

Cell pellet lysis was conducted using a SDS sample buffer (50 mM Tris/Cl, pH 6.8, 2% SDS, 10% glycerol, 4% beta-mercaptoethanol, and 0.02% bromophenol blue). Proteins were separated by 4–15% sodium dodecyl sulfate polyacrylamide gel electrophoresis (SDS-PAGE) gels and transferred using polyvinylidene fluoride (PVDF) membranes. Membranes were blocked in 5% nonfat milk for 30 mins and incubated with primary antibody overnight at 4 °C and washed three times with PBST (0.1% Tween 20 in PBS), followed by incubation with secondary antibody for 1 h at room temperature. Finally, immunoblot signals were detected using enhanced chemiluminescence.

### Immunoprecipitation

Cells were lysed on ice in TAP lysis buffer (20 mM Tris-HCL, pH7.4, 0.5% NP40, 150 mM NaCl, 1 mM NaF, 1 mM Na_3_VO_4_, 1 mM EDTA) supplemented with protease inhibitor cocktail for 30 min, followed by centrifuging for 15 min at 13,000 rpm. The cell lysate was incubated with antibody-conjugated beads for 5 h at 4 °C and then washed with TAP buffer. The bound proteins were analyzed by immunoblotting.

### Immunostaining, confocal microscopy

For immunofluorescence, cells were seeded on coverslips using 6-well plates and then fixed with 4% paraformaldehyde diluted in PBS at room temperature for 15 min and permeabilized with digitonin. Cells were blocked with 5% goat serum in PBS for 1 h at room temperature, and then incubated with primary antibodies overnight at 4 °C. After washing three times with PBS, cells were incubated with fluorescently labeled secondary antibodies (Alexa Fluor® 488, Alexa Fluor® 594, Cy3, or Alexa Fluor® 647) for 1.5 h at room temperature, re-washed, and mounted. Images were acquired with a confocal microscope (FV3000, Olympus) with a 100× (oil) objective. All images were processed using FluoView™ FV3000 software (Olympus).

### Tandem affinity purification and mass spectrometry

ZZ-VAMP8-Flag stable expressing HEK293T cell pellets were lysed in TAP buffer for 30 min on ice, and then centrifuged at 15,000 g for 30 min. The supernatant was incubated with IgG beads for 10 h at 4 °C. After centrifuging at 3,000 g for 15 min, the IgG beads were washed three times. The TEV protease was then incubated with IgG beads for 9 h. After incubation, the IgG beads were centrifuged and the supernatant was incubated with Flag beads. The pulled down proteins were eluted using Flag peptide for 3 h at 4 °C.

SDS-PAGE gel bands were destained and digested by trypsin at 37 °C for 16 h. The peptides were then dissolved in 0.1% TFA and dried using a SpeedVac lyophilizer. The samples were then resuspended with 0.1% formic acid for mass spectrometry (MS) analysis using a Thermo Fisher LTQ Orbitrap ETD mass spectrometer. A C18 column (1.8 mm, 0.15 × 1.00 mm) was used in the Thermo Fisher Easy-nLC 1000 high performance liquid chromatography (HPLC) system. Solvent A was aqueous (containing 0.1% formic acid) and solvent B was 100% acetonitrile. The elution gradient ranged from 4% to 18% over 182 min, then 18% to 90% over 13 min, at a flow rate of 300 nL/min. The MS/MS was acquired using higher-energy collision dissociation at 35% collision energy and mass resolution of 15,000. Raw MS files were analyzed using MaxQuant (version 1.5.2.8). The parameters used for data analysis included trypsin as the protease, with a maximum of two missed cleavages allowed. The mass tolerances for precursor and fragment ions were set to 20 ppm and 4.5 ppm, respectively. The minimum peptide length was set to six amino acids, with a maximum of two missed cleavages allowed. The false discovery rate (FDR) was set to 0.01 for both peptide and protein identification.

### Recombinant protein purification

Different GST-fused proteins were expressed in BL21 cells at 19 °C overnight after being induced by isopropyl-beta-D-thiogalactopyranoside (IPTG). Cell pellets were centrifuged at 4,000 rpm and lysed into *Escherichia coli* lysis buffer (20 mM Tris, pH 8.0, and 10% glycerol), followed by incubation with Glutathione Sepharose 4 Fast Flow beads (GE17-5132-01, GE Healthcare) overnight at 4 °C. They were then eluted with TEV cleavage buffer containing TEV protease. The purified proteins were further purified by gel filtration, then aliquoted and frozen in liquid nitrogen.

### In vitro affinity binding assay

Recombinant proteins were used to detect the interactions of VAMP8 WT and mutants with other SNARE proteins. GST-tagged proteins were incubated with GST beads for 1 h at 4 °C. Then, recombinant STX17, SNAP29 proteins were added to the system. After 1 h incubation, the GST-beads were centrifuged at 2,500 rpm and washed three times and the bound proteins were then boiled in SDS buffer, followed by SDS-PAGE and immunoblotting.

### In vitro kinase assay

Myc-tagged mTORC1 or mTORC1 Kinase dead mutant and HA-tagged Raptor were co-transfected into HEK293T cells. After 24-h transfection, cells were collected and lysed in TAP buffer. HA beads were incubated with the lysates for 5 h at 4 °C. Beads were washed three times before a kinase assay. For each in vitro kinase reaction, recombinant substrate and HA beads with kinase were incubated in a kinase buffer (20 mM HEPES at pH 7.4, 10 mM MgCl_2_, 5 μCi ATP) at 30 °C for 30 min, followed by SDS-PAGE and visualization by autoradiography.

### Protein expression and purification

Recombinant t- and v-SNARE proteins were expressed in *E. coli* strain BL21(DE3) and purified by nickel affinity chromatography. VAMP8 mutants were generated by site-directed mutagenesis and purified similarly to WT proteins. SNAREs were stored in a buffer containing 25 mM HEPES (pH 7.4), 400 mM KCl, 1% n-octyl-β-D-glucoside (OG), 10% glycerol, and 1 mM Dithiothreitol (DTT).

### Proteoliposome reconstitution and lipid mixing assays

All lipids were obtained from Avanti Polar Lipids Inc. For t-SNARE reconstitution, 1-palmitoyl-2-oleoyl-sn-glycero-3-phosphocholine (POPC), and 1-palmitoyl-2-oleoyl-sn-glycero-3-phosphoethanolamine (POPE) were mixed in a molar ratio of 80:20. For v-SNARE reconstitution, POPC, POPE, N-(7-nitro-2,1,3-benzoxadiazole-4-yl)-1,2-dipalmitoyl phosphatidylethanolamine (NBD-DPPE), and N-(Lissamine rhodamine B sulfonyl)-DPPE (rhodamine-DPPE) were mixed at a molar ratio of 80:17:1.5:1.5. SNARE proteoliposomes were prepared by detergent dilution and isolated on a Nycodenz (Axis-Shield) density gradient. Detergent was removed by overnight dialysis using Novagen dialysis tubes against the reconstitution buffer (25 mM HEPES [pH 7.4], 100 mM KCl, 10% glycerol, and 1 mM DTT).

A standard lipid-mixing reaction contained 5 μM t-SNARE and 1.5 μM v-SNARE. v-SNARE liposomes were labeled with NBD and rhodamine and were mixed with t-SNARE liposomes to initiate fusion. The fusion reactions were conducted in a 96-well microplate at 37 °C. The NBD-fluorescence (excitation: 460 nm; emission: 538 nm) was measured every two minutes in a BioTek Synergy HT microplate reader. At the end of the reaction, 10 μL of 10% CHAPSO was added to each sample. Fusion data were presented as the percentage of maximum fluorescence change.

### Mouse experiments

C57BL/6 J male mice (8-weeks-old) were obtained from Charles River Co. and acclimatized for 1 week. Temperature and humidity were monitored, and the mice were housed under a 12 h/12 h light/dark cycle. To investigate the role of VAMP8 phosphorylation, the indicated AAVs were packaged and intraperitoneally injected into mice. Four weeks post injection, the mice were used for experiments. All animal experimental protocols were approved by the Institutional Animal Care and Use Committee of Nanjing Agricultural University.

### Electron microscopy

Liver tissue from the mouse model was fixed with 2.5% glutaraldehyde in 0.1 M phosphate buffer, pH 7.4, overnight at 4 °C and washed with 0.1 M phosphate buffer, pH 7.4 three times, and then fixed with 2% aqueous osmium tetraoxide for 1.5 h. Samples were then dehydrated and embedded in epon 812 resin. Ultrathin sections were stained with 2% uranyl acetate and 0.3% lead citrate. Electron microscopy images were observed using an H-7650B (Hitachi) electron microscope.

### Oil red O and HE staining

Liver tissue from the indicated mouse models were fixed with 4% paraformaldehyde for 24 h at 4 °C. For oil red O staining, tissue sections were incubated in propylene glycol and stained with oil red O solution, followed by washing three times, and finally mounting with aqueous mounting solution. For hematoxylin and eosin (HE) staining, nuclei of tissue sections were stained with alum hematoxylin, washed three times with water, and then differentiated with acid alcohol, stained with eosin, dehydrated, and mounted with mounting buffer.

### Statistics and reproducibility

All the western blot and immunofluorescence assay was carried out at least three independent times with the same results.

### Reporting summary

Further information on research design is available in the Nature Research Reporting Summary linked to this article.

## Supplementary information

Supplementary Information

Reporting Summary

## Data Availability

The materials of this study are available from the corresponding author upon request. The mass spectrometry proteomics data have been deposited to the ProteomeXchange Consortium via the iProX partner repository with the dataset identifier PXD024642. Source data are provided with the paper.

## Source data

Source Data
