# Supplementary material for: mTOR-mediated phosphorylation of VAMP8 and SCFD1 regulates autophagosome maturation
Source: Nat Commun. 2021 Nov 16;12:6622. doi: 10.1038/s41467-021-26824-5 (PMC8595342; doi:10.1038/s41467-021-26824-5)

Supplementary Table 1

REAGENT or RESOURCE	SOURCE	IDENTIFIER
Antibodies		
Rabbit polyclonal anti-STX17 antibody	Sigma-Aldrich	Cat# HPA001204; RRID:AB_1080118
Rabbit monoclonal anti-VAMP8antibody	Abcam	Cat# ab76021; RRID:AB_1310798
Mouse monoclonal anti-Human SQSTM1 antibody	Novus	Cat#H00008878-M01; RRID:AB_548364
Mouse monoclonal anti- c-Myc (9E10) antibody	Santa Cruz	Cat# sc-40; RRID:AB_627268
Mouse monoclonal anti-Human LAMP-2 antibody	Santa Cruz	Cat# sc-18822; RRID:AB_626858
Rabbit polyclonal anti-Human HA antibody	Sigma-Aldrich	Cat# H6908; RRID: AB_260070
Rabbit polyclonal anti-LC3B antibody	Sigma-Aldrich	Cat#L7543; RRID:AB_796155
Rabbit polyclonal anti-Flag antibody	Sigma-Aldrich	Cat#F7425; RRID:AB_439687
Mouse monoclonal anti-Tubulin antibody	Sigma-Aldrich	Cat#T8328; RRID:AB_1844090
Rabbit polyclonal anti-SCFD1 antibody	Proteintech	Cat# 12569-1-AP; RRID:AB_2183266
Rabbit polyclonal anti-P70S6antibody	Cell Signaling Technology	Cat# 9202; RRID:AB_331676
Rabbit polyclonal anti-P70S6K(Thr389) antibody	Cell Signaling Technology	Cat# 9234; RRID:AB_2269803
Rabbit polyclonal anti-Acetylated-Lysine antibody	Cell Signaling Technology	Cat# 9441; RRID: AB_331805
Rabbit polyclonal anti-mTORC1 antibody	Cell Signaling Technology	Cat#2983; RRID:AB_2105622
Rabbit polyclonal anti-Raptor	Cell Signaling Technology	Cat#2280; RRID:AB_10694695
Chicken polyclonal anti-DDDDK tag antibody	Abcam	Cat# ab1170; RRID:AB_298495
Rabbit monoclonal anti-SNAP29antibody	Abcam	Cat#ab138500; RRID: AB_2687667
Chicken polyclonal anti- GFP antibody	Abcam	Cat# ab13970; RRID: AB_311908
Rabbit polyclonal anti-phosphoVAMP8	Homemade	Proteintech
Mouse monoclonal anti-phospho-Ser/Thr-Pro	Millipore	Cat#05-368; RRID: AB_309698
Goat anti-Mouse IgG (H+L), HRP	Proteintech	Cat# SA00001-1; RRID:AB_2722565
Goat anti-Rabbit IgG (H+L), HRP	Proteintech	Cat# SA00001-2; RRID: AB_2722564
Goat anti-Mouse IgG (H+L), Alexa 488	Thermo Fisher Scientific	Cat# A-11029; RRID:AB_138404
Goat anti-Rabbit IgG (H+L), Alexa 488	Thermo Fisher Scientific	Cat# A-11034; RRID:AB_2576217
Goat anti-Mouse IgG (H+L), Alexa 647	Thermo Fisher Scientific	Cat#A-21235; RRID:AB_2535804
Goat anti-Rabbit IgG (H+L), Alexa 647	Thermo Fisher Scientific	Cat#A-21245; RRID:AB_2535813
Goat anti-Mouse IgG (H+L), Cy3	Thermo Fisher Scientific	Cat#A10521; RRID:AB_2534030
Goat anti-Rabbit IgG (H+L), Cy3	Thermo Fisher Scientific	Cat# A10520; RRID:AB_2534029
Goat anti-Mouse IgG (H+L), 594	Thermo Fisher Scientific	Cat# A32742; RRID: AB_2762825
Goat anti-Rabbit IgG (H+L), 594	Thermo Fisher Scientific	Cat# A-11012; RRID:AB_141359
ANTI-Flag M2 Affinity Gel	Sigma-Aldrich	Cat#A2220

Anti-HA Affinity Gel	Sigma-Aldrich	Cat# A2095
Glutathione Sepharose 4 Fast Flow	GE Healthcare	Cat#GE17-5132-01
Bacterial and Virus Strains		
DH5a	ATCC	Cat# PTA-8019
BL21	Weidi Biotechnology	Cat#EC1001
Rosetta	Weidi Biotechnology	Cat# EC1010
AAV-Vector	Vigene biosciences	This paper
AAV-VAMP8	Vigene biosciences	This paper
AAV-VAMP8^{2A}	Vigene biosciences	This paper
AAV-VAMP8^{2D}	Vigene biosciences	This paper
Chemicals, Peptides, and Recombinant Proteins		
Chloroquine	Sigma-Aldrich	Cat# C6628; CAS: 50-63-5
Torin1	Sigma-Aldrich	Cat#475991; CAS 1222998-36-8
Lipofectamine 3000	Thermo Fisher Scientific	Cat#L3000015
Lipofectamine RNAiMAX	Thermo Fisher Scientific	Cat# 13778150
IPTG	Promega	Cat# V3955
EASYpack Protease Inhibitor Cocktail	Sigma-Aldrich	Cat# 5892970001
3 X Flag Peptide	Sigma-Aldrich	Cat# F4799
Trizol	Solarbio	Cat# M8018
VAMP8 recombinant proteins	Purified from *E. Coil*	homemade
SNAP29 recombinant proteins	Purified from *E. Coil*	homemade
STX17 recombinant proteins	Purified from *E. Coil*	homemade
TEV	Purified from *E. Coil*	homemade
Experimental Models: Cell Lines		
U2OS	ATCC	ATCC HTB-96
HEK293T	ATCC	ATCC CRL-321
Hela	ATCC	ATCC CCL-2
Experimental Models: Organisms/Strains		
Male C57BL/6 mice	Charles River	N/A
Oligonucleotides		
Raptor shRNA1: GATGAGGCTGATCTTACAG	This paper	N/A
Raptor shRNA2: ATCCTTAGCTCAGAGCTGG	This paper	N/A
VAMP8 siRNA1: AGGAAAUGAUCGUGUGCGGAACCU	This paper	N/A
VAMP8 siRNA2: GGCUCGAAAAUUCUGGUGGAAGAA	This paper	N/A
SCFD1 siRNA: GCUAUCUGUGAAGGAGCUATT	This paper	N/A
Recombinant DNA		
PTY-Flag-VAMP8	This paper	N/A
PTY-Flag-VAMP8^{2A}	This paper	N/A
PTY-Flag- VAMP8^{2D}	This paper	N/A

PMRX-GFP-VAMP8	This paper	N/A
PMRX-GFP-VAMP8^{2A}	This paper	N/A
PMRX-GFP- VAMP8^{2D}	This paper	N/A
PMRX-GFP-SNAP29	This paper	N/A
PMRX-GFP-STX17	This paper	N/A
pGEX4T-Flag-STX17	This paper	N/A
pGEX4T-Flag-SNP29	This paper	N/A
pGEX4T-Flag-VAMP8	This paper	N/A
pGEX4T-Flag-VAMP8^{2A}	This paper	N/A
pGEX4T-Flag- VAMP8^{2D}	This paper	N/A
Myc-mTORC1	Donated by Haixin Yuan	N/A
Myc-MTORC1 KD	Donated by Haixin Yuan	N/A
HA-Raptor	Donated by Haixin Yuan	N/A
Software and Algorithms		
ImageJ	ImageJ	https://imagej.nih.gov/ij/
GraphPad Prism	GraphPad	https://www.graphpad.com/
Olympus	Fluoview FV3000	N/A

Supplementary Figure 1

(A) MS-based identification of the VAMP8 phosphorylation sites.

(B) Identification of SCFD1 as a VAMP8-associated protein. Silver staining of the tandem affinity-purified Flag-VAMP8 and its associated proteins. The stable ZZ-VAMP8-Flag expressing HEK293T cell pellets were suspended in TAP buffer, followed by IgG bead binding and Flag bead binding.

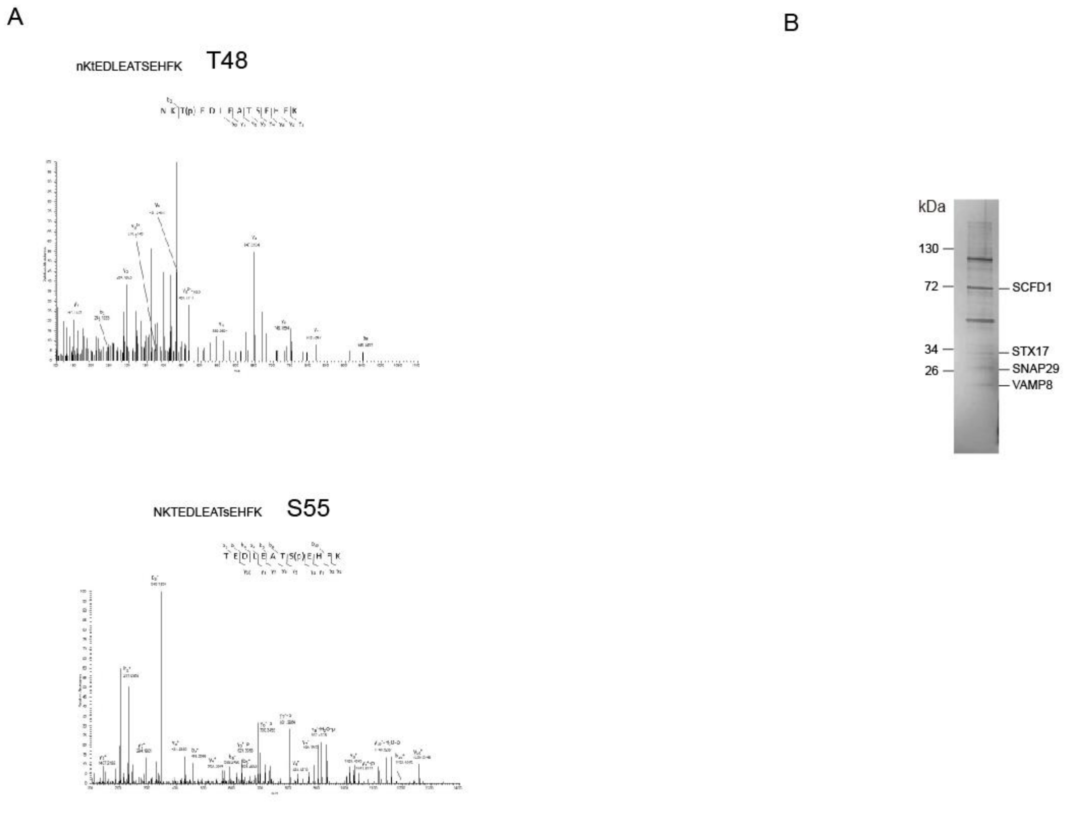

Supplementary Figure 2

Immunoblot analysis of WT Hela cells or U2OS cells stably expressing the VAMP8, VAMP8^{2A}, or VAMP8^{2D} proteins in Hela cells under OA treatment for the indicated times.

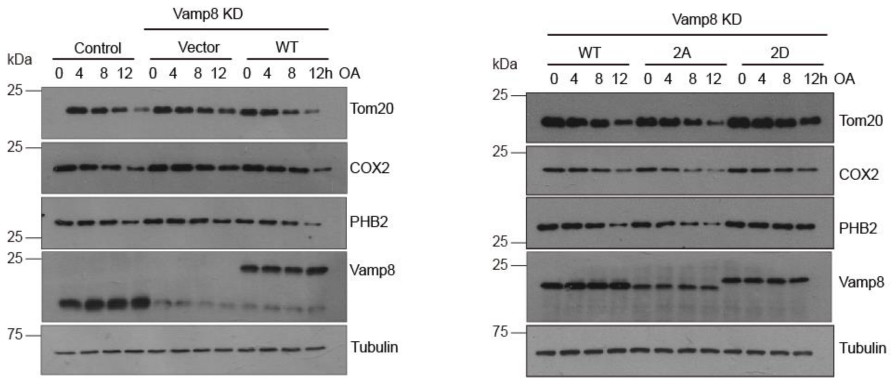

Supplementary Figure 3

Crystal structure analysis of VAMP8 48T location in an autophagy SNARE complex.

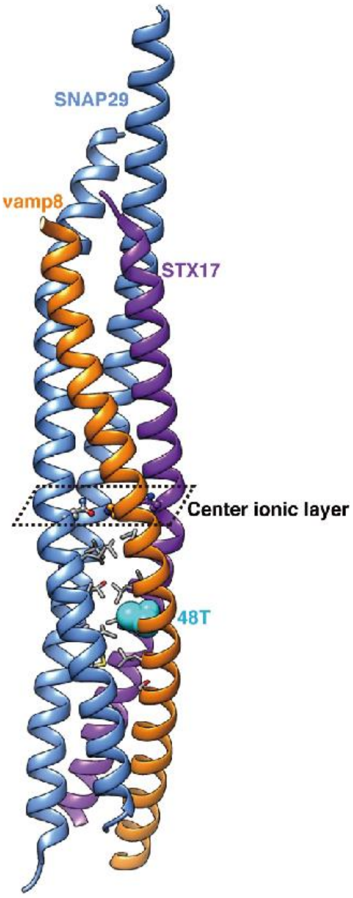

Supplementary Figure 4

(A) HEK293T cells transfected with Flag-STX17 or Flag-CD63. The interaction with endogenous SCFD1 was subsequently analyzed using anti-Flag immunoprecipitation.

(B) Co-IP of endogenous SCFD1 and Flag-STX17 or Flag-LAMP1.

(C) Co-IP of Flag-SCFD1 and endogenous STX17, VAMP8, or CD63.

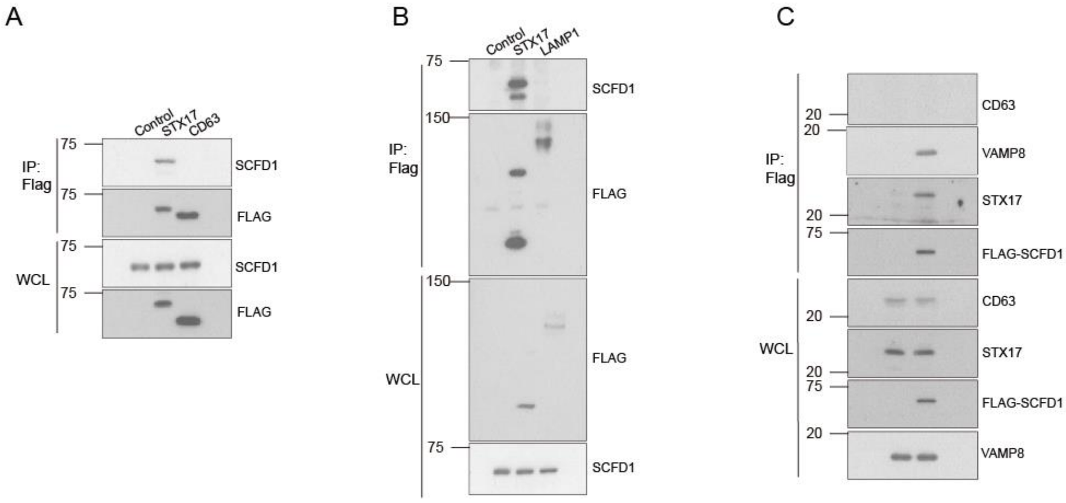

Supplementary Figure 5

Flag-tagged STX17, Flag-NTD, Flag-NTD, Flag-ΔNTD, Flag-SNARE, Flag-Δ SNARE, Flag-STX17TM, and Flag-STX17ΔTMD were co-expressed with GFP-SCFD1, and were subsequently analyzed using anti-Flag immunoprecipitation. STX17 without SNARE domain failed to undergo an interaction with SCFD1.

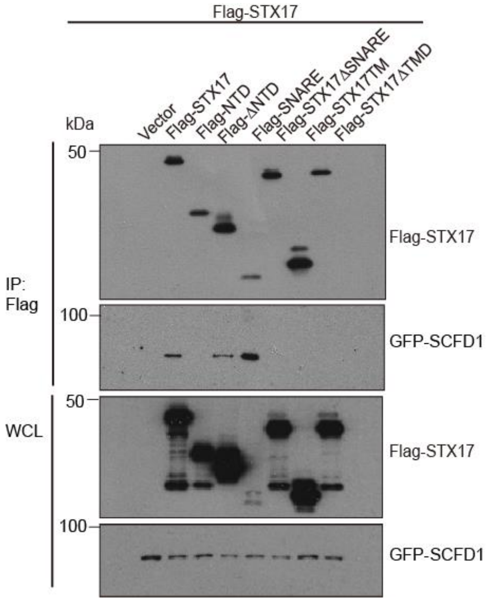

Supplement: Supplementary file 1 — Supplementary Information [file 41467_2021_26824_MOESM1_ESM.pdf]
