# Supplementary material for: mTOR-mediated phosphorylation of VAMP8 and SCFD1 regulates autophagosome maturation
Source: Nat Commun. 2021 Nov 16;12:6622. doi: 10.1038/s41467-021-26824-5 (PMC8595342; doi:10.1038/s41467-021-26824-5)
Supplement: Supplementary file 2 — Reporting Summary [file 41467_2021_26824_MOESM2_ESM.pdf]

nature research

Corresponding author(s): RONG LIU

Last updated by author(s): Oct. 7, 2021

# Reporting Summary

Nature Research wishes to improve the reproducibility of the work that we publish. This form provides structure for consistency and transparency in reporting. For further information on Nature Research policies, see our Editorial Policies and the Editorial Policy Checklist.

## Statistics

For all statistical analyses, confirm that the following items are present in the figure legend, table legend, main text, or Methods section.

| n/a | Confirmed | |
|---|---|---|
| ☐ | ☒ | The exact sample size ($n$) for each experimental group/condition, given as a discrete number and unit of measurement |
| ☐ | ☒ | A statement on whether measurements were taken from distinct samples or whether the same sample was measured repeatedly |
| ☐ | ☒ | The statistical test(s) used AND whether they are one- or two-sided<br>*Only common tests should be described solely by name; describe more complex techniques in the Methods section.* |
| ☐ | ☒ | A description of all covariates tested |
| ☐ | ☒ | A description of any assumptions or corrections, such as tests of normality and adjustment for multiple comparisons |
| ☐ | ☒ | A full description of the statistical parameters including central tendency (e.g. means) or other basic estimates (e.g. regression coefficient) AND variation (e.g. standard deviation) or associated estimates of uncertainty (e.g. confidence intervals) |
| ☐ | ☒ | For null hypothesis testing, the test statistic (e.g. $F$, $t$, $r$) with confidence intervals, effect sizes, degrees of freedom and $P$ value noted<br>*Give P values as exact values whenever suitable.* |
| ☒ | ☐ | For Bayesian analysis, information on the choice of priors and Markov chain Monte Carlo settings |
| ☒ | ☐ | For hierarchical and complex designs, identification of the appropriate level for tests and full reporting of outcomes |
| ☒ | ☐ | Estimates of effect sizes (e.g. Cohen's $d$, Pearson's $r$), indicating how they were calculated |

*Our web collection on statistics for biologists contains articles on many of the points above.*

## Software and code

Policy information about availability of computer code

| | |
|---|---|
| Data collection | Images were taken with the built-in softwares of Olympus FV3000 Microscope (version FV31S-SW). |
| Data analysis | Image J (version 1.43m),Prism(version 7.0e), |

For manuscripts utilizing custom algorithms or software that are central to the research but not yet described in published literature, software must be made available to editors and reviewers. We strongly encourage code deposition in a community repository (e.g. GitHub). See the Nature Research guidelines for submitting code & software for further information.

## Data

Policy information about availability of data

All manuscripts must include a data availability statement. This statement should provide the following information, where applicable:

- Accession codes, unique identifiers, or web links for publicly available datasets
- A list of figures that have associated raw data
- A description of any restrictions on data availability

All data supporting the findings of this study are provided in the Article and its Supplementary Information, or from the corresponding author on reasonable request. Source data are provided with this paper.

# Field-specific reporting

Please select the one below that is the best fit for your research. If you are not sure, read the appropriate sections before making your selection.

☒ Life sciences ☐ Behavioural & social sciences ☐ Ecological, evolutionary & environmental sciences

For a reference copy of the document with all sections, see nature.com/documents/nr-reporting-summary-flat.pdf

# Life sciences study design

All studies must disclose on these points even when the disclosure is negative.

| | |
|---|---|
| Sample size | Sample size estimates has been performed on previous experience to obtain statistical significance and reproducibility. For in vitro experiments such as Western blot , at least three samples were used per group for minimal statistics requirements. For in vivo studies, the sample size was determined to be sufficient to obtain the statistical difference between groups. |
| Data exclusions | All data produced are included. |
| Replication | All experiments underlying main conclusions of this study have been successfully replicated multiple times and corroborated by several models. All the western blot and image assay were carried out at least three independent times with the same results. |
| Randomization | Samples and organisms were randomly allocated to experimental groups. No specific randomization protocol has been used. Mice were age and sex matched. |
| Blinding | No specific blinding was applied since all experiments were assigned into groups including relevalant controls and analysis was done objectively and without bias. |

# Behavioural & social sciences study design

All studies must disclose on these points even when the disclosure is negative.

| | |
|---|---|
| Study description | *Briefly describe the study type including whether data are quantitative, qualitative, or mixed-methods (e.g. qualitative cross-sectional, quantitative experimental, mixed-methods case study).* |
| Research sample | *State the research sample (e.g. Harvard university undergraduates, villagers in rural India) and provide relevant demographic information (e.g. age, sex) and indicate whether the sample is representative. Provide a rationale for the study sample chosen. For studies involving existing datasets, please describe the dataset and source.* |
| Sampling strategy | *Describe the sampling procedure (e.g. random, snowball, stratified, convenience). Describe the statistical methods that were used to predetermine sample size OR if no sample-size calculation was performed, describe how sample sizes were chosen and provide a rationale for why these sample sizes are sufficient. For qualitative data, please indicate whether data saturation was considered, and what criteria were used to decide that no further sampling was needed.* |
| Data collection | *Provide details about the data collection procedure, including the instruments or devices used to record the data (e.g. pen and paper, computer, eye tracker, video or audio equipment) whether anyone was present besides the participant(s) and the researcher, and whether the researcher was blind to experimental condition and/or the study hypothesis during data collection.* |
| Timing | *Indicate the start and stop dates of data collection. If there is a gap between collection periods, state the dates for each sample cohort.* |
| Data exclusions | *If no data were excluded from the analyses, state so OR if data were excluded, provide the exact number of exclusions and the rationale behind them, indicating whether exclusion criteria were pre-established.* |
| Non-participation | *State how many participants dropped out/declined participation and the reason(s) given OR provide response rate OR state that no participants dropped out/declined participation.* |
| Randomization | *If participants were not allocated into experimental groups, state so OR describe how participants were allocated to groups, and if allocation was not random, describe how covariates were controlled.* |

# Ecological, evolutionary & environmental sciences study design

All studies must disclose on these points even when the disclosure is negative.

| | |
|---|---|
| Study description | *Briefly describe the study. For quantitative data include treatment factors and interactions, design structure (e.g. factorial, nested, hierarchical), nature and number of experimental units and replicates.* |

| | |
|---|---|
| Research sample | *Describe the research sample (e.g. a group of tagged Passer domesticus, all Stenocereus thurberi within Organ Pipe Cactus National Monument), and provide a rationale for the sample choice. When relevant, describe the organism taxa, source, sex, age range and any manipulations. State what population the sample is meant to represent when applicable. For studies involving existing datasets, describe the data and its source.* |
| Sampling strategy | *Note the sampling procedure. Describe the statistical methods that were used to predetermine sample size OR if no sample-size calculation was performed, describe how sample sizes were chosen and provide a rationale for why these sample sizes are sufficient.* |
| Data collection | *Describe the data collection procedure, including who recorded the data and how.* |
| Timing and spatial scale | *Indicate the start and stop dates of data collection, noting the frequency and periodicity of sampling and providing a rationale for these choices. If there is a gap between collection periods, state the dates for each sample cohort. Specify the spatial scale from which the data are taken* |
| Data exclusions | *If no data were excluded from the analyses, state so OR if data were excluded, describe the exclusions and the rationale behind them, indicating whether exclusion criteria were pre-established.* |
| Reproducibility | *Describe the measures taken to verify the reproducibility of experimental findings. For each experiment, note whether any attempts to repeat the experiment failed OR state that all attempts to repeat the experiment were successful.* |
| Randomization | *Describe how samples/organisms/participants were allocated into groups. If allocation was not random, describe how covariates were controlled. If this is not relevant to your study, explain why.* |
| Blinding | *Describe the extent of blinding used during data acquisition and analysis. If blinding was not possible, describe why OR explain why blinding was not relevant to your study.* |

Did the study involve field work? ☐ Yes ☐ No

## Field work, collection and transport

| | |
|---|---|
| Field conditions | *Describe the study conditions for field work, providing relevant parameters (e.g. temperature, rainfall).* |
| Location | *State the location of the sampling or experiment, providing relevant parameters (e.g. latitude and longitude, elevation, water depth).* |
| Access & import/export | *Describe the efforts you have made to access habitats and to collect and import/export your samples in a responsible manner and in compliance with local, national and international laws, noting any permits that were obtained (give the name of the issuing authority, the date of issue, and any identifying information).* |
| Disturbance | *Describe any disturbance caused by the study and how it was minimized.* |

# Reporting for specific materials, systems and methods

We require information from authors about some types of materials, experimental systems and methods used in many studies. Here, indicate whether each material, system or method listed is relevant to your study. If you are not sure if a list item applies to your research, read the appropriate section before selecting a response.

### Materials & experimental systems

| n/a | Involved in the study | |
|---|---|---|
| ☐ | ☒ | Antibodies |
| ☐ | ☒ | Eukaryotic cell lines |
| ☒ | ☐ | Palaeontology and archaeology |
| ☐ | ☒ | Animals and other organisms |
| ☒ | ☐ | Human research participants |
| ☒ | ☐ | Clinical data |
| ☒ | ☐ | Dual use research of concern |

### Methods

| n/a | Involved in the study | |
|---|---|---|
| ☒ | ☐ | ChIP-seq |
| ☒ | ☐ | Flow cytometry |
| ☒ | ☐ | MRI-based neuroimaging |

## Antibodies

Antibodies used

Rabbit polyclonal anti-STX17 antibody Sigma-Aldrich Cat# HPA001204; RRID:AB_1080118(1:1000)

Rabbit monoclonal anti-VAMP8antibody Abcam Cat# ab76021; RRID:AB_1310798(EP2629Y, 1:1000)

Mouse monoclonal anti-Human SQSTM1 antibody Novus Cat#H00008878-M01; RRID:AB_548364(2C11,1:1000)

Mouse monoclonal anti- c-Myc (9E10) antibody Santa Cruz Cat# sc-40; RRID:AB_627268(9E10,1:1000)

Mouse monoclonal anti-Human LAMP-2 antibody Santa Cruz Cat# sc-18822; RRID:AB_626858(H4B4,1:1000)

| | | |
|---|---|---|
| Rabbit polyclonal anti-Human HA antibody | Sigma-Aldrich | Cat# H6908; RRID: AB_260070(1:1000) |
| Rabbit polyclonal anti-LC3B antibody | Sigma-Aldrich | Cat#L7543; RRID:AB_796155(1:5000) |
| Rabbit polyclonal anti-FLAG antibody | Sigma-Aldrich | Cat#F7425; RRID:AB_439687(1:10000) |
| Mouse monoclonal anti-Tubulin antibody | Sigma-Aldrich | Cat#T8328; RRID:AB_1844090(AA2,1:5000) |
| Rabbit polyclonal anti-SCFD1 antibody | Proteintech | Cat# 12569-1-AP; RRID:AB_2183266(1:1000) |
| Rabbit polyclonal anti-P70S6antibody | Cell Signaling Technology | Cat# 9202; RRID:AB_331676(1:1000) |
| Rabbit polyclonal anti-P70S6K(Thr389) antibody | Cell Signaling Technology | Cat# 9234; RRID:AB_2269803(1:1000) |
| Rabbit polyclonal anti-Acetylated-Lysine antibody | Cell Signaling Technology | Cat# 9441; RRID: AB_331805(1:1000) |
| Rabbit polyclonal anti-mTORC1 antibody | Cell Signaling Technology | Cat#2983; RRID:AB_2105622(1:1000) |
| Rabbit polyclonal anti-Raptor | Cell Signaling Technology | Cat#2280; RRID:AB_10694695(1:1000) |
| Chicken polyclonal anti-DDDDK tagantibody | Abcam | Cat# ab1170; RRID:AB_298495(1:1000) |
| Rabbit monoclonal anti-SNAP29antibody | Abcam | Cat#ab138500; RRID: AB_2687667(EPR9199,1:1000) |
| Chicken polyclonal anti- GFP antibody | Abcam | Cat# ab13970; RRID: AB_311908(1:1000) |
| Rabbit polyclonal anti-phosphoVAMP8 | Homemade | Proteintech(1:1000) |
| Mouse monoclonal anti-phospho-Ser/Thr-Pro | Millipore | Cat#05-368; RRID: AB_309698(mpm2,1:1000) |
| Goat anti-Mouse IgG (H+L), HRP | Proteintech | Cat# SA00001-1; RRID:AB_2722565(1:1000) |
| Goat anti-Rabbit IgG (H+L), HRP | Proteintech | Cat# SA00001-2; RRID: AB_2722564(1:1000) |
| Goat anti-Mouse IgG (H+L), Alexa 488 | Thermo Fisher Scientific | Cat# A-11029; RRID:AB_138404(1:1000) |
| Goat anti-Rabbit IgG (H+L), Alexa 488 | Thermo Fisher Scientific | Cat# A-11034; RRID:AB_2576217(1:1000) |
| Goat anti-Mouse IgG (H+L), Alexa 647 | Thermo Fisher Scientific | Cat#A-21235; RRID:AB_2535804(1:1000) |
| Goat anti-Rabbit IgG (H+L), Alexa 647 | Thermo Fisher Scientific | Cat#A-21245; RRID:AB_2535813(1:1000) |
| Goat anti-Mouse IgG (H+L), Cy3 | Thermo Fisher Scientific | Cat#A10521; RRID:AB_2534030(1:1000) |
| Goat anti-Rabbit IgG (H+L), Cy3 | Thermo Fisher Scientific | Cat# A10520; RRID:AB_2534029(1:1000) |
| Goat anti-Mouse IgG (H+L), 594 | Thermo Fisher Scientific | Cat# A32742; RRID: AB_2762825(1:1000) |
| Goat anti-Rabbit IgG (H+L), 594 | Thermo Fisher Scientific | Cat# A-11012; RRID:AB_141359(1:1000) |

Validation

For all the antibodies, we carried out western blot according to the method on the company's website, and detected whether the band size met the expectation with molecular weight marker, and added appropriate positive control and negative control. For SCFD1, STX17, VAMP8, and so on, we added siRNA knockdown verification. Antibodies purchased from Cell SignalingTechnology and Abcam were validated as per their website stating "Antibody signal is measured in model systems with known presence/absence of target signal. Includes wild-type vs. genetic knockout, targeted induction or silencing."

## Eukaryotic cell lines

Policy information about cell lines

Cell line source(s): U2OS, Hela and HEK293T cells were purchased from ATCC.

Authentication: All cell lines were authenticated by providers (STR profiling).

Mycoplasma contamination: Cells used for all experiments were mycoplasma negative.

Commonly misidentified lines (See ICLAC register): None of commonly misidentified cell lines has been used.

## Animals and other organisms

Policy information about studies involving animals; ARRIVE guidelines recommended for reporting animal research

Laboratory animals: C57BL6J mice, 8-12 weeks old, male. All mice were cultured in suitable temperature and humidity environment, and fed with sufficient water and food.（25℃， suitable humidity (typically 50%)， 12 hour dark/light cycle）

| | |
|---|---|
| Wild animals | None |
| Field-collected samples | None |
| Ethics oversight | the Animal Care and Use Committee of the animal facility at Nanjing Agricultural University |

Note that full information on the approval of the study protocol must also be provided in the manuscript.
